# Piloting PrePex for Adult and Adolescent Male Circumcision in South Africa – Pain Is an Issue

**DOI:** 10.1371/journal.pone.0138755

**Published:** 2015-09-25

**Authors:** Limakatso Lebina, Noah Taruberekera, Minja Milovanovic, Karin Hatzold, Miriam Mhazo, Cynthia Nhlapo, Nkeko Tshabangu, Mmatsie Manentsa, Victoria Kazangarare, Millicent Makola, Scott Billy, Neil Martinson

**Affiliations:** 1 Perinatal HIV Research Unit, Faculty of Health Sciences, University of the Witwatersrand, Johannesburg, South Africa; 2 Population Services International, Johannesburg, South Africa; 3 Population Services International, Harare, Zimbabwe; 4 Society for Family Health, Johannesburg, South Africa; 5 Johns Hopkins University School of Medicine, Baltimore, United States of America; Cardiff University, UNITED KINGDOM

## Abstract

**Background:**

The World Health Organisation and the Joint United Nations Programme on HIV/AIDS have recommended the scale-up of Medical Male Circumcision (MMC) in countries with high HIV and low MMC prevalence. PrePex device circumcision is proposed as an alternate method for scaling up MMC.

**Objective:**

Evaluate safety and feasibility of PrePex in South Africa.

**Design:**

A multisite prospective cohort PrePex study in adults and adolescents at three MMC clinics. Participants were followed-up 8 times, up to 56 days after PrePex placement.

**Results:**

In total, 398 PrePex circumcisions were performed (315 adults and 83 adolescents) their median ages were 26 (IQR: 22–30) and 16 years (IQR: 15–17), respectively. The median time for device placement across both groups was 6 minutes (IQR: 5–9) with the leading PrePex sizes being B (30%) and C (35%) for adults (18–45 years), and A (31%) and B (38%) for adolescents (14–17 years). Additional sizes (size 12–20) were rarely used, even in the younger age group. Pain of device application was minimal but that of removal was severe. However, described pain abated rapidly and almost no pain was reported 1 hour after removal. The Adverse Events rate were experienced by 2.7% (11/398) of all participants, three of which were serious (2 displacements and 1 self-removal requiring prompt surgery). None of the Adverse Events required hospitalization. The majority of participants returned to work within a day of device placement.

**Conclusion:**

Our study shows that PrePex is a safe MMC method, for males 14 years and above. PrePex circumcision had a similar adverse event rate to that reported for surgical MMC, but device removal caused high levels of pain, which subsided rapidly.

## Introduction

The World Health Organisation (WHO) and the Joint United Nations Programme on HIV/AIDS (USAID) have recommended the scale-up of voluntary Medical Male Circumcision (MMC) in countries with a high HIV prevalence rate [1] following the findings of 3 clinical trials that confirmed the preventative benefits of circumcision in HIV acquisition [2–4]. The fourteen countries in Eastern and Southern Africa that were prioritised in 2007 have actually adopted the rapid scale-up of MMC as an HIV prevention strategy [1].

South Africa has a high HIV prevalence (12.2%) with 6.4 million HIV infected people [5]. In response to that the South African Government in 2010 introduced the voluntary MMC policy and programme with the aim of reaching 80% (4.3 million) of HIV negative men between the age of 15–49 years by 2015 [5, 6] and by end of March 2014, 1.4 million men had been circumcised [7]. However, the programme has been experiencing problems with: acceptability; shortages of supplies and personnel; poor quality of services provided; and inadequate monitoring of adverse events [5, 8].

There has been an increase in research on device-based circumcisions with the hope that these devices will make the circumcision procedure quicker, safer and require less-skilled personnel, yet remain as acceptable as surgical circumcision [9]. Several devices (PrePex, Shang Ring and Unicirc) have been evaluated [10–12]. In June 2013, PrePex became the first non-surgical device that was prequalified by the WHO for circumcision in adults [13]. PrePex is promoted as being quicker and less painful than surgical circumcision; and that it requires no anaesthesia, suturing or haemostasis and therefore should be able to be safely used by healthcare workers with mid-level surgical skills [13]. This is the first study evaluating acceptability and feasibility of PrePex in South Africa.

This study’s objective, therefore, was to obtain data to inform the implementation of PrePex circumcision in South Africa. The study was conducted according to the WHO framework for clinical evaluation of devices for male circumcision [14].

## Methods

We conducted a prospective cohort study of adult (18–49 years) and adolescent (14–17 years) males who were circumcised with the PrePex device. The study was conducted in two high volume male circumcision clinics (Witbank Hospital and Tsakane Clinic) and a HIV wellness and MMC clinic (Zuzimpilo clinic). The sub-study on the <18 year old participants was only done in Tsakane and Zuzimpilo clinics. Witbank MMC site, located in the eMalahleni Municipality in the Mpumalanga Province, with a population of 395,466 [15] was established in May 2012 and had done a cumulative total of 12,294 circumcisions by July 2014. The HIV prevalence for pregnant women in the area for 2011 was 36.1%, for the general population it was 22.5% [15]. Tsakane clinic was established in January 2012 and had done a cumulative total of 16,564 circumcisions by July 2014. The clinic is located in the Ekurhuleni Municipality in Gauteng Province [16]. The population HIV prevalence in Ekurhuleni was estimated to be 14.3% in 2012 [5]. Zuzimpilo clinic is located in downtown Johannesburg and has provided over 6000 patients with care and treatment for chronic diseases mainly HIV. Medical male circumcision was started at Zuzimpilo in December 2009 mainly using the surgical forceps guided method, and 1200 circumcisions had been done by mid-2014. Johannesburg Metro has an HIV prevalence of 11.1% [5].

All three clinics are staffed with doctors and/or clinical associates, nurses, counsellors and administration personnel. All clinicians who used the PrePex device received standardised training and certification. Certification required the trainee to have done at least 15 PrePex placements and 10 device removals under the supervision of a PrePex Master Trainer.

Prior to circumcision, HIV status was established either through counselling and testing by trained counsellors at the clinic or receiving a written copy of HIV results from the referring centre. The clinics promote circumcision and emphasise abstinence for six weeks following the procedure and encourage condom use.

Males were considered eligible for the study if they were: voluntarily seeking MMC at one of the study sites; willing to have a circumcision by PrePex device; were between the ages of 18–45 years for adults and 14–17 for adolescents; HIV seronegative; participants penises had to fit a sizing template for one of the PrePex device sizes (PrePex device has five adult sizes (size A-E) and five smaller sizes (size 12–20). The smaller Prepex sizes were made specifically for adolescents and are not part of the prequalified sizes A-E for adults. Males with medical and penile anatomical conditions that made them unsuitable for PrePex device or out-patient MMC were excluded from the study but were offered a forceps-guided circumcision, or referred for further care if necessary.

All three sites participating in the study followed similar study procedures and data collection. Males (aged 14–45 years) were recruited from the surrounding areas, within a 25km radius, for MMC. All MMC clients received information, during individual and group sessions, on forceps guided MMC method, which was the standard surgical procedure, and also on PrePex device MMC. Clients then selected the circumcision method they preferred. Individual consent for PrePex was obtained from those interested in participating in the study. The consent included items that specifically addressed not tampering with, or having sex while the device was placed and abstaining from sex for at least six weeks after removal of the device. The parent or guardian of participants <18 years of age had to provide written informed consent for both circumcision and participation in this study and the participants were also required to sign assent forms.

On the day of placement: baseline information was collected; size was measured and PrePex device fitted following guidelines provided by manufacturer; the participants were instructed not to touch or remove the device, to avoid vigorous contact (bath normally) and not have sex or masturbate while the device was in place and for six weeks after removal. Participants were also given a pamphlet on post-application care. Photographs of the device in-situ were taken on the day of placement and at each follow-up visit. A visual analogue scale (VAS) was used to rate pain from 0 (very happy, no hurt) to 10 (hurts as much as you can imagine) at placement, during telephonic follow-up (day 1 and 3 post-placement), immediately prior to removal, immediately after removal, and at 30 and 60 minutes thereafter.

Following device placement all participants on discharge were dispensed 20 tablets of 500mg Paracetamol to take orally when required. Device removal was scheduled 7 days after placement. Seven additional follow-up visits (Telephone day 1 and 3, clinic visit day 14, 21, 42, 49 and 56 post-placement) were scheduled to assess pain, discomfort, wound healing, resumption of normal activities and adverse events. Participants were encouraged to return to the clinic in case of any problems or concerns at any time outside of the scheduled visits. Those who missed their visits were contacted telephonically; and a clinic team member conducted a home visit if no contact was made. Participants were declared a loss to follow-up one month after a scheduled visits and if we could not reach them. All adverse events reported were classified according to the PSI/COSECS Adverse Event Action Guide as this study was done within a programme and only moderate and serious adverse events have been included in this analysis [17]. Minor adverse events (AE) were classified as requiring little or no intervention, moderate AEs required active treatment and severe AEs required hospitalization or surgery. Some adverse events that required no treatment were categorized as moderate. As a result, a committee of the most experienced team members was formed to review all adverse events and reclassify according to protocol.

## Data Analysis

The sample size was informed by the WHO Framework for Clinical Evaluation of Devices for Adult Male Circumcision [14]. All tests were conducted at the significance level of 0.05. Descriptive statistics (frequency, median, interquartile ranges, percentages) were used to describe quantitative variables. The primary outcomes assessed included: pain, discomfort and time required for placement and removal; adverse events and time to complete healing. Time to complete healing was analysed using Kaplan Meir survival analyses.

The study was approved by the University of the Witwatersrand’s Human Research Ethics Committee (Medical) and the research committee of the Provincial Department of Health in Mpumalanga.

## Results

### Baseline Characteristics of Men Being Circumcised

**Fig 1:**
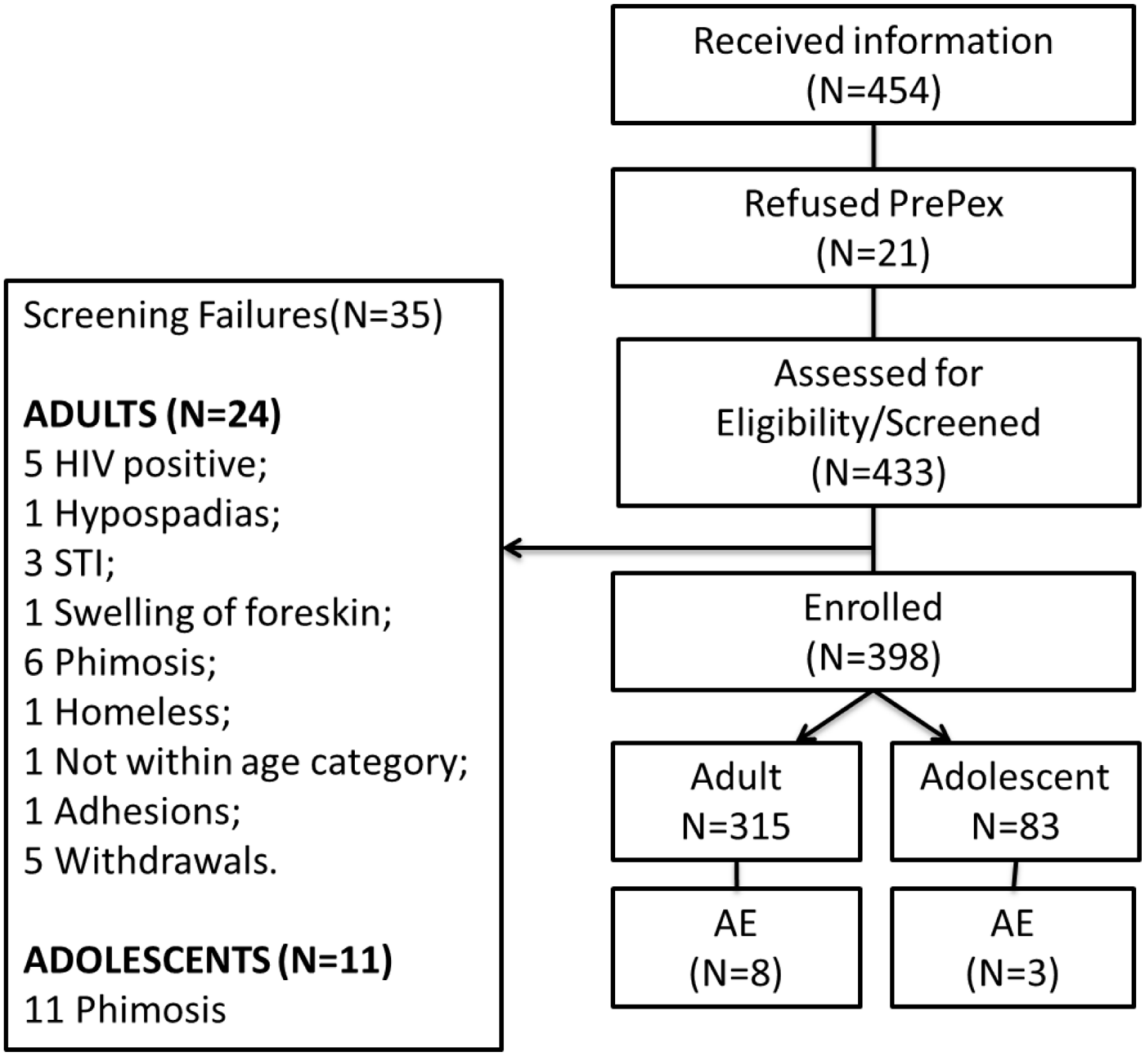
Flow chart of total PrePex participants assessed, screened and enrolled. A Total of 454 participants received information and 21 refused to participate. Among the 433 participants that were assessed for eligibility 35 (24 adults and 11 adolescents) were not suitable therefore a total of 398 (315 adults and 83 adolescents) participants were enrolled. We observed 8 adverse events in adults and 3 in adolescents.

In the nine months from August 2013, a total of 454 male adults and adolescents received PrePex information. Of those that received information about PrePex, 21 refused PrePex circumcision during the consenting process; 433 were consented and then screened, of whom 35 failed the screening process: 24/339 (7%) were adults and 11/94 (11.7%) were adolescents. The main reason for screening failure in adolescents was phimosis (11 participants had phimosis) while in adults 5 were HIV positive, 6 phimosis, 5 withdrew after consenting and others had STI or anatomical abnormalities (Fig 1). Adolescents had a significantly higher rate of exclusion due to anatomical abnormalities, mostly phimosis (p<0.05). In total, 398 participants were enrolled into the study; 315 adults aged 18–45 years (including five men 46–48 years of age that were inadvertently recruited), and 83 adolescents aged 14–17 years (Fig 1). The median age of adult men who had PrePex circumcision was 26 years (IQR: 22–30) and that of adolescents was 16 years (IQR: 15–17) (Table 1).

**Table 1:** Baseline Characteristics of PrePex Participants.

	Adult	Adolescent
**Number of participants**	315	83
**Age (In Years)**	26 (IQR:22–30)	16 (IQR:15–16.5)
**Income (ZAR)**	2750 (IQR:0–4800)	
**Education (N = 295)**	No education: 1% (2/295)	
	Some Primary school: 3% (9/295)	
	Completed Primary school: 2% (5/295)	
	Grade 8–10: 19% (56/295)	
	Grade 11–12: 76% (223/295)	
**Language (N = 294)**	Zulu: 57% (167/294)	
	SeSotho: 9% (25/294)	
	Swati: 6% (19/294)	
	Xhosa: 3% (8/294)	
**PrePex Sizes**	A: 9% (30/313)	A: 31% (25/82)
	B: 30% (94/313)	B: 38% (31/82)
	C: 35% (108/313)	C: 16% (13/82)
	D: 19% (60/313)	D: 1% (1/82)
	E: 7% (21/313)	E: 2% (2/82)
		Other: 12% (10/82)
**Other PrePex Sizes**		Size 14: 10% (1/10)
		Size 16: 20% (2/10)
		Size 18: 40% (4/10)
		Size 20: 30% (3/10)
**Application time (Minutes)**	7 (IQR: 5–9)	6 (IQR:5–9)

### PrePex Placement

Virtually all participants (92.5%) had the PrePex device applied on the day of screening. Overall, the application took a median of 6 minutes (IQR: 5–9) from cleaning of genitals, to leaving the operating table with the device applied.

**Fig 2:**
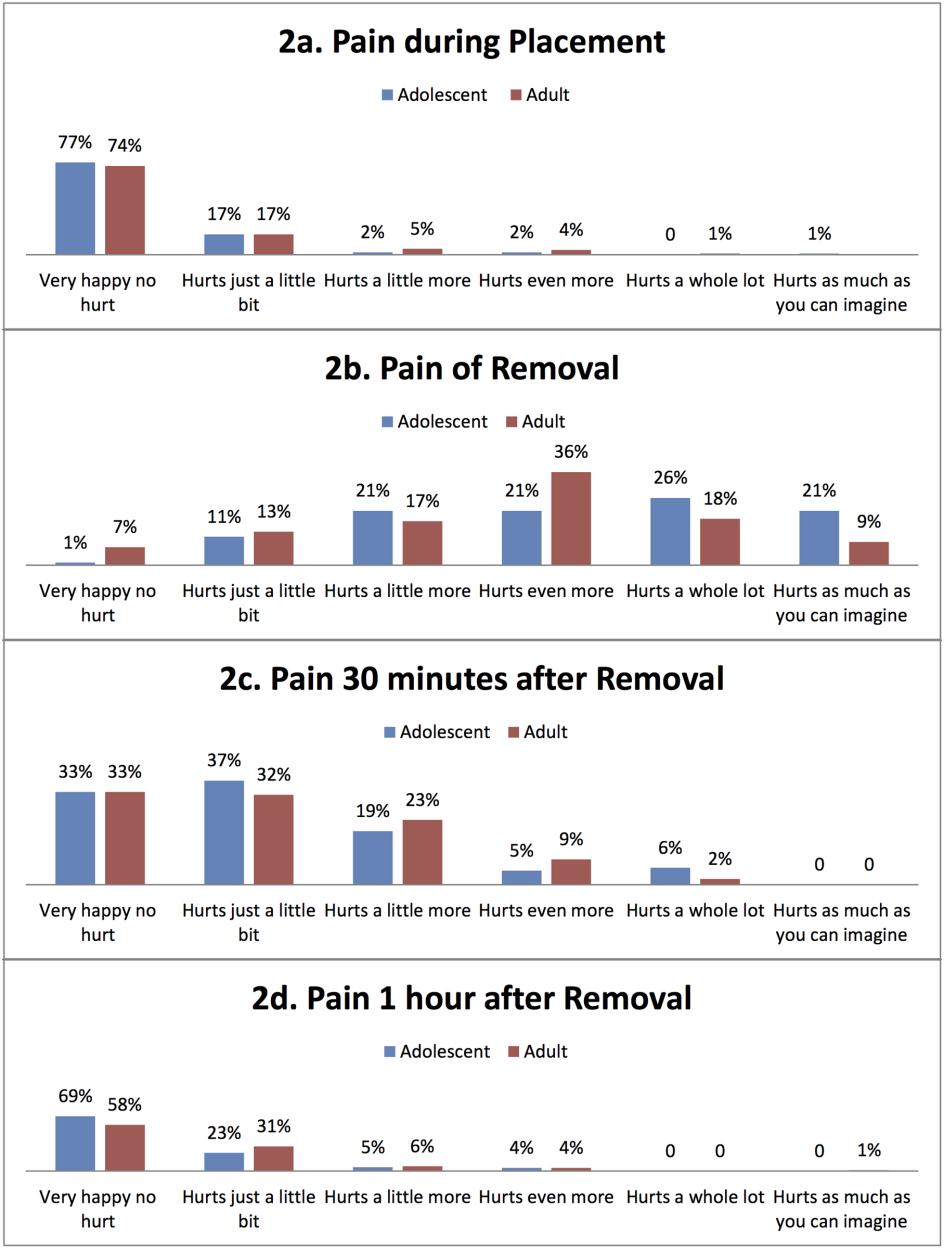
Pain during PrePex procedure. Adult and adolescent participants had little or no pain during the placement procedure. However, during the removal procedure they experienced moderate to severe pain which abated rapidly and virtually no pain was reported 1 hour after removal.

Most PrePex applications (77%: 300/392) were performed by doctors or clinical associates and one quarter (23%: 92/392) were performed by nurses. No data was available on 6 participants. Nurses had a median PrePex application time of 7 minutes (IQR: 5–10) while doctors took 6 minutes (IQR: 5–9). Although 23% of adolescents (age 14–17 years) and 26% of the adults (18–45 years) reported experiencing mild pain during placements, the majority reported no pain (Fig 2a).

### Device Size Distribution

In adults, the leading PrePex sizes were B and C (65%) and in adolescents they were A and B (69%) (Table 1). Only 12% (10/82) of adolescents used the special sizes (14–20) intended for adolescents. There were no participants that did not fit one of the available sizes.

### PrePex Removal

PrePex removals were scheduled for day 7 post-application but 8 adolescents had PrePex removed on day 6, and 21 adults had their removal between day 4 and 6 after application, while three other adults had removals between day 0 and 2(1 self-removal and two displacements. The main reasons for early removal included severe pain and difficulty voiding. All participants that had their device removed early continued with the scheduled visits with no additional unexpected events. Prior to PrePex device removal, participants were asked whether they experienced any non-pain related discomfort while wearing the device with both adults (21%: 65/311) and adolescents (18%: 15/83) experiencing some discomfort (smell, tightness and itchiness).

Participants were also asked whether they experienced anything unexpected during the PrePex circumcision that they did not like. Twenty three (adults and adolescents) experienced an unexpected event while wearing the device and after removal. Thirteen participants reported a bad odour associated with the device on and other common complaints include swelling and itchiness. Few participants reported having used additional pain medication (2.5%: 10/398) in the week while the device was in-situ.

PrePex device removal was more painful than device application. Adolescents reported higher pain during the removal procedure than adults with 47% (38/82) of adolescents having had a pain rating between 8–10 compared to 27% (85/309) of adults (p = 0.001) (Fig 2b). However, reported pain abated rapidly and virtually no pain was reported 1 hour after removal (Fig 2c and 2d).

### Resumption of Normal and Sexual Activities

Following PrePex device placement, 98% of adolescents (81/83) and 85% of adults (269/315) had returned to work or school within one day. Of the adults who did not return to work within one day, their median resumption time was 3 days (IQR: 2–7).

Thirty from 288 (10.4%) adult participants had resumed sexual activity prior to day 56 follow-up. One adult had reported resumption of sexual activity prior to device removal (masturbation on day 1) and experienced a device displacement. Median resumption of sexual activity in adult males following removal of PrePex device was 43.5 days (IQR: 35–52); 10 reported resumption of sexual activity at 30–49 days and 19 between 50–60 days. Of the 29 adult participants: 1 participant masturbated at day 35 post-placement; and 28 had vaginal intercourse with 20 (71%) having used condoms and 8 (29%) did not use condoms. No participants reported bleeding during sexual activity. No adolescents (aged 14–17 years) reported resuming or initiating sexual activity throughout the duration of the study.

### Time to Healing

**Fig 3:**
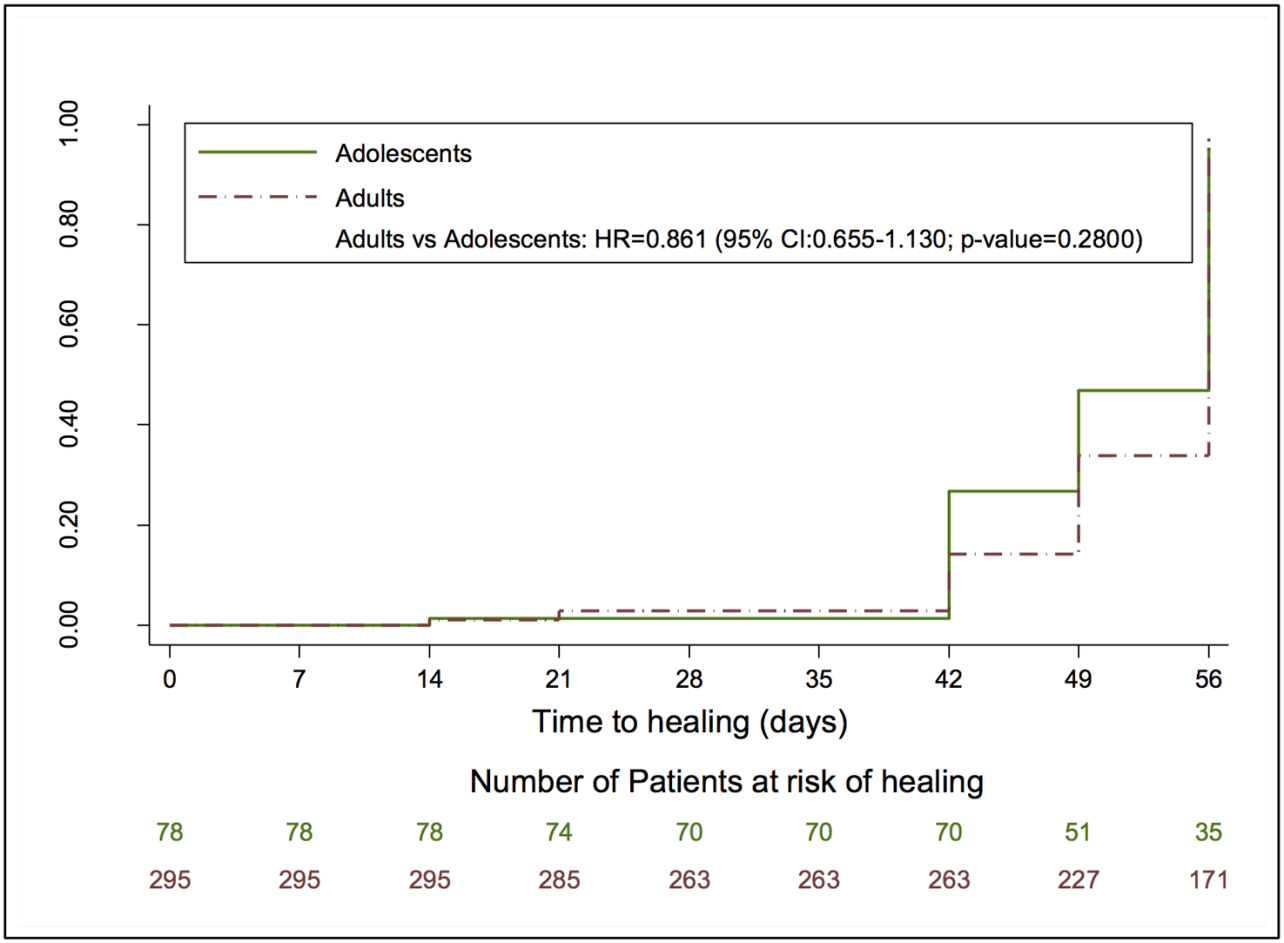
Kaplan Meier Survival Analysis of time to healing after application by adults and Adolescents. At the day 42 (post-application) review, 15% (39/263) adults and 27% (19/70) adolescents were healed. Of the 171 adults who were assessed on day 56 review, 164 (96%) were considered healed. For adolescents, 91% (32/35) were healed on day 56 review.

We were able to report healing time in 95% (78/82) of adolescents and 94% (295/315) of adults. At the day 42 (post-application) review, 15% (39/263) adults and 27% (19/70) adolescents were healed. A participant was classified as healed if the wound was completely closed and there was no pain or discomfort experienced. Of the 171 adults who were assessed on day 56 review, 164 (96%) were considered healed. For adolescents, 91% (32/35) were healed on day 56 review (Fig 3).

### Safety and Adverse Events

The overall adverse event rate, (excluding minor adverse events) was 2.7% (11/398) (Table 2). Three of the adverse events were classified as serious (2 displacements and 1 self-removal) because they required surgical circumcision however; all procedures were done within the circumcision clinics. The self-removal occurred 8 hours after device placement. There were no serious adverse events that required hospitalisation or were life-threatening. Minor adverse events were predominantly: obstructed urine flow caused by the leather-like necrotic foreskin obstructing the urethral meatus on day 4–5 and localised wound sepsis occurring two weeks post-removal. There were also no reported cases of tetanus. During the course of the study, all participants were provided with detailed explanation on how to prevent the urinary obstruction (disturbance of urine flow) during the counselling sessions. The participants were instructed to expose the head of the glans by gently retracting the foreskin after urination prior to necrosis setting in. When comparing between medical doctor and nurse operators, the adverse event rate was similar: 2.7% for doctors (8/300) and 3.3% for nurses (3/92) (p = 0.7642).

**Table 2:** Each moderate and serious adverse event with PrePex circumcision.

Individual Adverse Event	Age Category	Days following Placement	Treatment	Outcome
**ADULT**				
Bleeding	Adult	7	Compression	Resolved
Infection	Adult	28	Oral Antibiotics	Resolved
Infection	Adult	28	Oral Antibiotics	Resolved
Displacement or self-removal	Adult	2	Surgical Circumcision	Resolved
Displacement or self-removal	Adult	1	Surgical Circumcision	Resolved
Wound Dehiscence	Adult	18	Dressings	Delayed wound healing
Obstruction to void with UTI Symptoms	Adult	5	Opening with forceps and antibiotics	Resolved
Displacement or self-removal	Adult	0	Surgical Circumcision	Resolved
**ADOLESCENT**				
Infection	Adolescent	17	Antibiotics	Resolved
Bleeding	Adolescent	9	Compression	Resolved
Scrotal swelling and generalized oedema	Adolescent	47	Treatment from Urology Department at referral hospital	Resolved

The two device displacements and one self-removal had an occurrence rate of 0.8% (Table 2). The two displacements occurred on days 1 and 2 post-placement with the exact cause of displacement being unclear. Self-removal of PrePex device occurred on day 0 (placement) but participant only returned to the clinic on day 7, with swelling of the foreskin, for his check-up. The participant reported that he removed the device after masturbation. The two displacements and one self-removal were treated at the site with a modified dorsal slit circumcision.

### Loss to Follow-Up

The rate of loss to follow-up, post-removal, was 9% (35/391). Of those that were a loss to follow-up 66% (23/35) were adults (18–45 years) and 34% (12/35) were adolescents (14–17 years). The main reason for being declared a loss to follow-up was that clinic staff was unable to contact the participants or staff did make contact (either telephonically or through home visits) but participants never returned to the clinics.

## Discussion

The PrePex device appears to be safe for use in MMC clinics in South Africa and can be safely placed and removed by non-physician providers. Our study provides further insights into the use of this device. Firstly, severe pain (8–10 rating) is experienced by the vast majority of patients when the device is removed. Although we did not assess this, the pain at removal may be mitigated by analgesia or local anaesthetic administered prior to removal. Secondly, we identified an unexpected adverse event of urinary obstruction caused by closing down of the necrotic foreskin over the urethral meatus. Thirdly, we showed that in 10.4% of adults, sexual activity takes place prior to healing—possibly putting these patients at high risk of acquiring HIV.

Not all the participants who were screened were suitable for PrePex circumcision and 8% could not be enrolled because of anatomical abnormalities (phimosis, hypospadias). This is similar to previous studies which reported 6.5%-10% screening failures that were excluded from PrePex [18–19].

The median placement time in this study was at the higher end of what has been reported in other studies [20–21]. In our study, placement time was measured from the start of genital cleaning to the time when the patient left the operating bed; other published studies measured duration of procedure from application of cream to cutting of the verification thread and several studies did not define the measure [20]. The placement time we report is similar to the time required to place a Shang Ring Device [10, 22–23]. Minimal pain during placement of PrePex and no complications recorded are comparable to what other studies have reported on the PrePex placement procedure [20]. In a 2013 study by Mutabazi et al., it was reported that 95% (496/518) of participants described a pain level of 1 during placement [11].

The removal of the device was scheduled for day 7 post-placement however, there were 28 participants who returned to have the device removed before day 7, whereas in Galukande et al., 95% of participants returned for removal at 5–7 days post-placement [18]. The main challenge with removal was severe, short-lived pain, which is similar to other PrePex studies and the Shang Ring removal where the reported pain lasts about 2 minutes and required no intervention [10–11, 18, 20].

In this study and others, circumcision with PrePex had minimal interference with work and daily activities with participants reporting that they returned to work on the same day of placement or the following day [10–11, 20]. Of high concern is resumption of sexual activity prior to complete healing which occurred in 30 adults in this study—similar to the 49 reported by Fedlum et al. [20]. The healing time we report with PrePex (56 days post-placement) appears to be longer than other studies in patients surgically circumcised (4–6 weeks) [24] and longer than that reported for the Shang Ring, 4–6 weeks [10, 21–22], other PrePex studies [11] and Unicirc (4 weeks) [12]. Of concern is that unprotected sexual activity prior to healing may result in higher risk of acquiring HIV [25].

Previously reported rates of moderate and severe adverse events with PrePex ranged from 1% to 4% [11], comparable to that of surgical circumcision [2–4] and similar to the 2.7% reported in this study. Reported adverse events with Shang Ring range from 2–7% [22–23]. Device displacements and self-removals were infrequent in this and other PrePex studies [18, 11] and all participants had surgical circumcision as part of management. This highlights the importance of the need for relatively close access to a facility that can provide a surgical circumcision within a PrePex Device MMC program [13]. No Self-removal or displacement has been reported with the Shang Ring device [23].

This study has several limitations. Firstly, our adolescent sample size was small and we were limited to the age group of 14–17 years. Although there was extensive training for providers, assessment of healing and adverse events may have differed between sites. The use of the PSI/COSECS Adverse Event Action Guide for programs could have resulted in underreporting of adverse events, especially minor ones. Finally, losses to follow-up in the final visit were high. The day 49 visit in particular had the highest non-attendance similar to other studies [20–21].

## Conclusion

Our results suggest that PrePex placement is a relatively pain free procedure which, in this group of participants, no adverse events were observed during placement procedure. Device removal caused severe pain, and although transient, we feel this should be addressed. The Adverse Events rate were experienced by 2.7% (11/398) of all participants, three of which were serious (2 displacements and 1 self-removal requiring prompt surgery). None of the Adverse Events required hospitalization. Adult PrePex sizes were suitable for most of the adolescents but higher rates of ineligibility due to phimosis may limit scale-up in this group. PrePex is a safe procedure that can easily be incorporated into high volume male circumcision clinics in South Africa. In light of the longer healing time required for PrePex device circumcision and the self-reported early resumption of sexual activities in some participants, more studies are needed to assess the risk of HIV transmission in the immediate post-operative period.
